# The Decision to Engage Cognitive Control Is Driven by Expected Reward-Value: Neural and Behavioral Evidence

**DOI:** 10.1371/journal.pone.0051637

**Published:** 2012-12-19

**Authors:** Matthew L. Dixon, Kalina Christoff

**Affiliations:** 1 Department of Psychology, University of British Columbia, Vancouver, British Columbia, Canada; 2 Department of Psychiatry, University of British Columbia, Vancouver, British Columbia, Canada; Radboud University Nijmegen, The Netherlands

## Abstract

Cognitive control is a fundamental skill reflecting the active use of task-rules to guide behavior and suppress inappropriate automatic responses. Prior work has traditionally used paradigms in which subjects are told when to engage cognitive control. Thus, surprisingly little is known about the factors that influence individuals' initial decision of whether or not to act in a reflective, rule-based manner. To examine this, we took three classic cognitive control tasks (Stroop, Wisconsin Card Sorting Task, Go/No-Go task) and created novel ‘free-choice’ versions in which human subjects were free to select an automatic, pre-potent action, or an action requiring rule-based cognitive control, and earned varying amounts of money based on their choices. Our findings demonstrated that subjects' decision to engage cognitive control was driven by an explicit representation of monetary rewards expected to be obtained from rule-use. Subjects rarely engaged cognitive control when the expected outcome was of equal or lesser value as compared to the value of the automatic response, but frequently engaged cognitive control when it was expected to yield a larger monetary outcome. Additionally, we exploited fMRI-adaptation to show that the lateral prefrontal cortex (LPFC) represents associations between rules and expected reward outcomes. Together, these findings suggest that individuals are more likely to act in a reflective, rule-based manner when they expect that it will result in a desired outcome. Thus, choosing to exert cognitive control is not simply a matter of reason and willpower, but rather, conforms to standard mechanisms of value-based decision making. Finally, in contrast to current models of LPFC function, our results suggest that the LPFC plays a direct role in representing motivational incentives.

## Introduction

Everyday life involves constant decision making―what to eat, what to wear, who to talk to, what to say, etc. Abundant work has examined the neurocognitive mechanisms of decision making, and a common value-based framework has emerged, and suggests quite simply, that decisions are made by estimating the value of each option and then selecting the option with the higher expected value [1]. The orbitofrontal cortex (OFC) plays an important role in this process by representing the relationship between different options and expected motivational outcomes [2], [3], [4]. To date, most studies have utilized simple decision making paradigms, for example offering a choice between two different food options. As such, little is known about more complex decision making involving explicit rules for behavior. Consider a scenario in which a boss asks an employee to prepare a presentation summarizing the company's recent financial progress. This task requires cognitive control―actively holding in mind task rules in order to make an appropriate response (and suppressing automatic, pre-potent responses when necessary). What determines whether the employee will follow through and decide to actively hold in mind a set of rules for completing this task instead of going for a coffee break or doing something else that does not require cognitive control?

The traditional perspective is that the capacity for reflective, rule-based behavior is largely based on cold cognitive processes such as reason, knowledge of social standards, self-monitoring, and willpower, all of which serve to suppress the influence of desires that provoke unwanted behaviors [5], [6], [7]. However, this idea, predicated on an antithesis between reason and desire (or motivational processing more broadly), does not satisfactorily address the question of *why* individuals engage cognitive control in the first place. We follow Baumeister and Vohs [8] and Fujita [9] in suggesting that motivation is a crucial element of any decision, including those involving self-regulation and rule-based behavior. The proposal here, is that the decision to engage cognitive control is based on value-based mechanisms just like the decision of which fruit to eat with lunch. Thus, in our example, we suggest that the decision to act in a deliberate, rule-based manner and complete the proposal is dependent on the expectation that this will lead to a desired outcome (e.g., praise from the boss; a monthly paycheck).

It is now firmly established that cognitive control processes are influenced by motivational incentives [10], [11], [12], [13], [14], [15], [16], [17], and recent work has additionally shown that individuals engage cognitive control during decision making, e.g., choosing a healthy over a tasty food option [18], a larger, delayed reward over a smaller, immediate reward [19], and choosing to promote fairness at the cost of immediate financial benefit [20], [21]. However, prior work has not directly examined the factors underlying the *initial decision* to guide behavior based on explicit rules for action.

Our hypothesis suggests that a critical computation underlying the decision to engage cognitive control is forming associations between task-rules and expected motivational outcomes. While the OFC is critical for representing associations between expected outcomes and simple options (e.g., a visual object) [2], [3], [4], we hypothesized that the lateral prefrontal cortex (LPFC) may be crucial in the case of rule-outcome associations. The LPFC has been traditionally considered a purely “cognitive” region that supports rules for behaviour, and is not involved in motivational processes. However, accumulating evidence is more consistent with the idea that the LPFC does in fact play a direct role in motivational processes. First, the LPFC shares anatomical connections with both rule-related areas (posterior parietal, lateral temporal, and pre-motor cortices) and motivation-related areas (orbitofrontal cortex, rostral anterior cingulate, and insula) (for an overview see [22]). Second, electrophysiological and neuroimaging studies have demonstrated that the LPFC is often active during reward anticipation and receipt [23], [24], [25], [26], [27], [28]. Finally, recent neuroeconomic studies have revealed a direct correspondence between LPFC activity and the subjective value assigned to choice options [29], preference for temporally delayed rewards [19], [26], [30], decision making involving risk [31], [32], tracking action-outcome history [33], and a decision making strategy that focuses on maximizing the overall probability of winning money [34].

In Experiment 1, we modified three classic measures of cognitive control [Stroop, Wisconsin Card Sorting Task (WCST), and the Go/No-Go task] to provide a simple laboratory measure of decision making about rule-based behavior. We examined whether the decision to select a deliberate, rule-based action rather than an automatic pre-potent action is influenced by expected motivational outcomes. In Experiment 2, we used functional magnetic resonance imaging (fMRI) to look for neural evidence of rule-outcome associations. In particular, we took advantage of fMRI-adaptation, which has been widely used to directly examine the specific information represented by different brain regions [35], [36]. fMRI-adaptation is based on the fact that if a brain region represents a given piece of information, it will show a change in the magnitude of activation when that information is repeated as compared to when it is presented for the first time. Repeated relative to novel information often elicits a smaller neural response (repetition suppression), although in some cases a greater response is observed (repetition enhancement). fMRI-adaptation is most commonly used in studies of visual processing, but has also been used to examine the neural basis of stimulus-response learning [37], mirror neurons [38], semantic decision making [39], theory of mind [40] and rule representation [41]. Using a 2×2 factorial design in which the factors were rules (novel versus repeated) and reward outcome (novel versus repeated), we examined whether the LPFC shows fMRI-adaptation when there is repetition of a specific rule-outcome pairing, relative to when the pairing is novel.

## Results

### Behavioral Results

Subjects performed three different tasks (Stroop task, WCST, and Go/No-Go task) with the same structure. First, during a training period, an automatic pre-potent response was established by having subjects respond to the stimuli in one way over the course of many trials (e.g., Stroop: respond based on the word meaning). Second, there was a free-choice decision making period during which subjects had the option of selecting the automatic, pre-potent response, or an alternative response requiring cognitive control (e.g., Stroop: responding based on the ink colour now requires active maintenance of the task rules to overcome the pre-potent tendency to respond based on the word meaning). Prior to each mini-block of four decision making trials, subjects saw a screen indicating the amount of money that could be earned for selecting each response type (e.g., Stroop: word meaning = 25¢, ink color = 50¢). There was no feedback after choices; thus, decisions were most likely driven by an explicit representation of the expected monetary outcomes.

**Figure 1:**
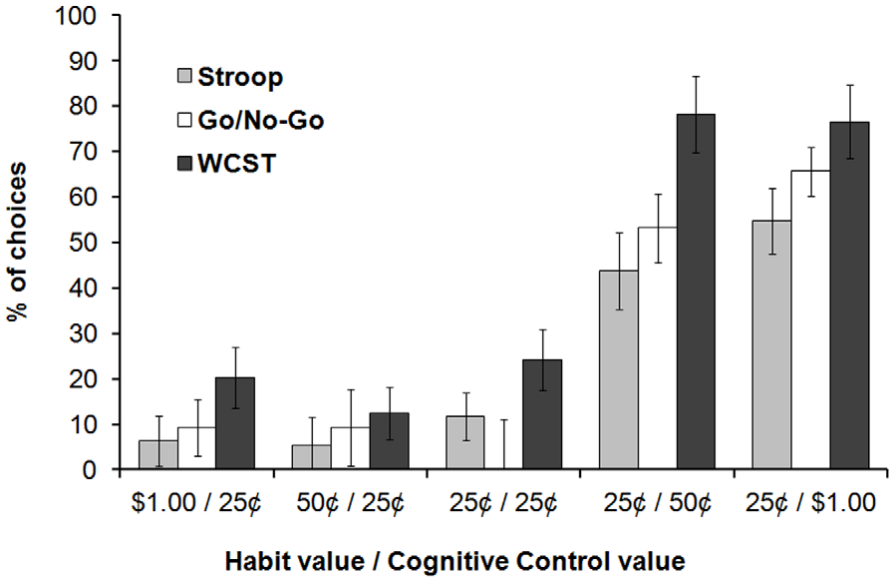
Behavioral Results. Percentage (%) of choices in which cognitive control was selected during the free-choice period as a function of expected monetary rewards. Error bars represent one (within-subject) standard error of the mean based on Loftus and Masson [69].

The results demonstrated that reaction times were faster when subjects selected the automatic response relative to the cognitive control-based response during the free-choice period, suggesting that the training period indeed led to a pre-potent response (paired t-test: Stroop: *p* = .059; WCST: *p*<.001; RTs could not be compared for the Go/No-Go task given that the cognitive control response was in fact withholding a response). Figure 1 illustrates the percentage of cognitive control responses during the free-choice period as a function of expected monetary rewards. Separate one-way repeated measures ANOVAs with expected reward amount as the independent variable indicated that for each task, subjects' decisions during the free-choice period were robustly influenced by the size of the expected monetary rewards for each response type [Stroop: *F*(4, 60) = 15.28, *p*<.001; WCST: *F*(4, 60) = 24.93, *p*<.001; Go/No-Go: *F*(4, 60) = 17.39, *p*<.001]. Subjects rarely engaged cognitive control when the expected monetary reward was equal to, or less than the expected monetary reward for the automatic, pre-potent response. In contrast, subjects frequently engaged cognitive control when it was expected to yield the larger payoff (i.e., 25¢/50 ¢ and 25¢/$1.00 conditions) (Figure 1). These findings provide direct evidence that the decision to engage cognitive control is driven by explicit expectations of motivational outcomes that will result from rule-use.

Many subjects remarked that the WCST was the easiest task, whereas the Stroop task was the most difficult, and interestingly, decisions were influenced by this apparent difference in task difficulty. A 2 (task: Stroop vs WCST) ×5 (expected reward amount) repeated measures ANOVA revealed that subjects were more likely to engage cognitive control in the WCST compared to the Stroop task [main effect of task: *F*(1, 15) = 5.68, *p* = .031], especially when engaging cognitive control was the more rewarding option [task x condition interaction: *F*(4, 60) = 2.58, *p* = .046] (Figure 1). This result could reflect a greater number of unsuccessful attempts at engaging cognitive control in the Stroop, or greater intentional selection of the automatic response. To differentiate, we performed an analysis at the level of mini-blocks. Subjects were aware that earning money was contingent on correct performance across each trial of a given mini-block. Therefore, selection of the cognitive control response on 3/4 trials would suggest one incorrect response during that mini-block, rather than intentional selection of the automatic response. Conversely, greater selection of the automatic response in the Stroop task would be reflected in a greater number of mini-blocks in which subjects selected the automatic response on 3/4 or 4/4 trials. This latter scenario is what we found [main effect of task: *F*(1, 15) = 7.34, *p* = .016], suggesting that the difference between the Stroop and WCST was driven by greater selection of the automatic response in the Stroop task.

### Neuroimaging Results

Having established that the decision to engage cognitive control is based on expected motivational outcomes, we next looked for evidence that the LPFC represents associations between rules and expected outcomes. Such associations should be a critical neural computation underlying the decision process. Although recent work has shown that motivational incentives modulate cognitive control related activation in the LPFC (e.g., [10], [11], it is assumed that this reflects an amplified representation of just the task rules [16]. However, it is just as possible that this pattern actually reflects a representation of the motivational outcome in relation to the rules. Thus, prior work has been unable to determine the specific nature of the information represented by the LPFC. To circumvent this interpretational limitation inherent to standard paradigms, we took advantage of fMRI-adaptation, a widely used as a tool for directly examining the specific information represented by different brain regions [35], [36].

**Figure 2:**
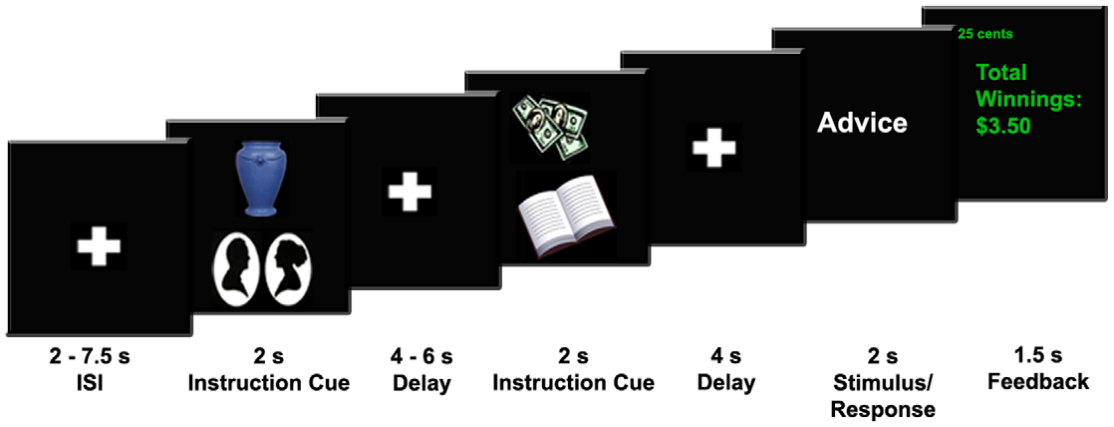
Trial Structure for the fMRI Experiment. After a variable duration fixation cross, an instruction cue signaled the currently relevant rules (profile of faces = male/female rule; book = abstract/concrete rule) and whether or not to expect a monetary reward (blue vase = no money; bills = 25¢). This was followed by a variable duration delay period and then a word or face stimulus, during which subjects made a button response. Finally, a screen revealed whether money had been earned on that trial and cumulative winnings. On key trials, a second instruction cue appeared before the stimulus. Across the two instruction cues, we varied whether there was repetition of the rules, expected reward, both, or neither.

In our paradigm (see Material and Methods and Figure 2), each trial started with an instruction cue that indicated one of two rules to use (male/female face discrimination, versus abstract/concrete word discrimination), and also indicated one of two expected motivational outcomes (25¢ monetary reward, versus no monetary reward). Following presentation of the instruction cue, subjects made a button response to a face or word stimulus. The key feature of the task is that on certain trials, a second instruction cue appeared prior to the stimulus, and relative to the first instruction cue, we manipulated whether there was repetition of the rules, repetition of the outcome, repetition of the rule-outcome pairing, or presentation of a novel rule-outcome pairing. (Although this task differs in surface features from the behavioral tasks, it shares the core cognitive control requirement of active maintenance of task rules due to the constant switching of rules from trial to trial. Moreover, this task was designed to minimize response and perceptual conflict, thus allowing us to examine the neural representation of rule-outcome associations in the absence of potential confounding variables.)

To examine fMRI-adaptation, we analyzed activation during the second instruction cue according to a 2 (rules: novel versus repeated) ×2 (outcome: novel versus repeated) factorial design. If the LPFC shows an interaction effect, demonstrating fMRI-adaptation when there is repetition of a specific rule-outcome pairing, but not when there is repetition of just the rules alone or just the outcome alone, then this would provide compelling evidence that the LPFC represents associations between rules and outcomes. fMRI-adaptation could manifest in two ways: 1) smaller activation for a repeated rule-outcome pairing relative to a novel pairing (repetition suppression), which could result from lower processing demands when re-activating a recently experienced rule-outcome pairing; or 2) greater activation for a repeated rule-outcome pairing (repetition enhancement), which could result from a reinforced expectation that a particular rule-outcome pairing would be used on that trial. For our purposes, the direction of the fMRI-adaptation effect was not important. Rather, the important question was whether we would observe fMRI-adaptation selectively during the repeated rule-outcome pairing condition.

#### Behavioral Results

Consistent with the idea that subjects were representing the relationship between rules and expected motivational outcomes, subjects were faster to respond during single cue trials when money was available to be won (*M*_money_ = 857.70, *SD* = 58.77) as compared to when no money was available (*M*_no_ _money_ = 898.83, *SD* = 92.54) [*t*(14) = 2.82, *p* = .014; two-tailed]. There was no difference in accuracy (*M*_money_ = 98.13%, *SD* = 3.66% vs *M*_no_ _money_ = 96.87%, *SD* = 2.95%) (*p*>.3). This incentive effect suggests that subjects were paying attention to the instruction cues and using them to prepare for each trial.

#### Validation of the fMRI-Adaptation Paradigm

We first examined the main effects of our 2×2 factorial design to validate our paradigm. Canonical regions associated with reward processing were expected to show fMRI-adaptation when reward information alone was repeated, and canonical regions associated with rule processing were expected to show fMRI-adaptation when rule information alone was repeated. Indeed, consistent with prior work [29], [30], [42], repetition of the expected monetary reward outcome was associated with repetition suppression (i.e., a smaller BOLD response) in the rostral (pregenual) anterior cingulate cortex (rACC) extending into the anterior mid-cingulate cortex (aMCC), bilateral nucleus accumbens (NAcc), globus pallidus, bilateral anterior insula, caudal OFC, posterior cingulate cortex (PCC), near the mid-brain dopaminergic nuclei, and left inferior frontal gyrus (Table S1; *Z*>2.57, *p*<.05 FWE cluster-corrected).

Consistent with prior work examining rule processing [41], [43], [44], repetition of the rules was associated with repetition suppression in the left inferior frontal gyrus (IFG; pars triangularis), left ventral premotor cortex extending into the posterior IFG (pars opercularis), supplementary motor area, aMCC, cerebellum, and left lateral temporal cortex including the posterior middle temporal gyrus (Table S2; *Z*>2.57, *p*<.05 FWE cluster-corrected). Repetition enhancement for repeated rules was observed in the left rostrolateral prefrontal cortex, left posterior middle frontal gyrus, and bilateral inferior parietal lobule. Together these findings demonstrate that key reward related areas exhibited fMRI-adaptation when just the motivational outcome was repeated, and key rule related areas exhibited fMRI-adaptation when just the rules were repeated.

#### The LPFC Represents Rule-Outcome Associations

**Figure 3:**
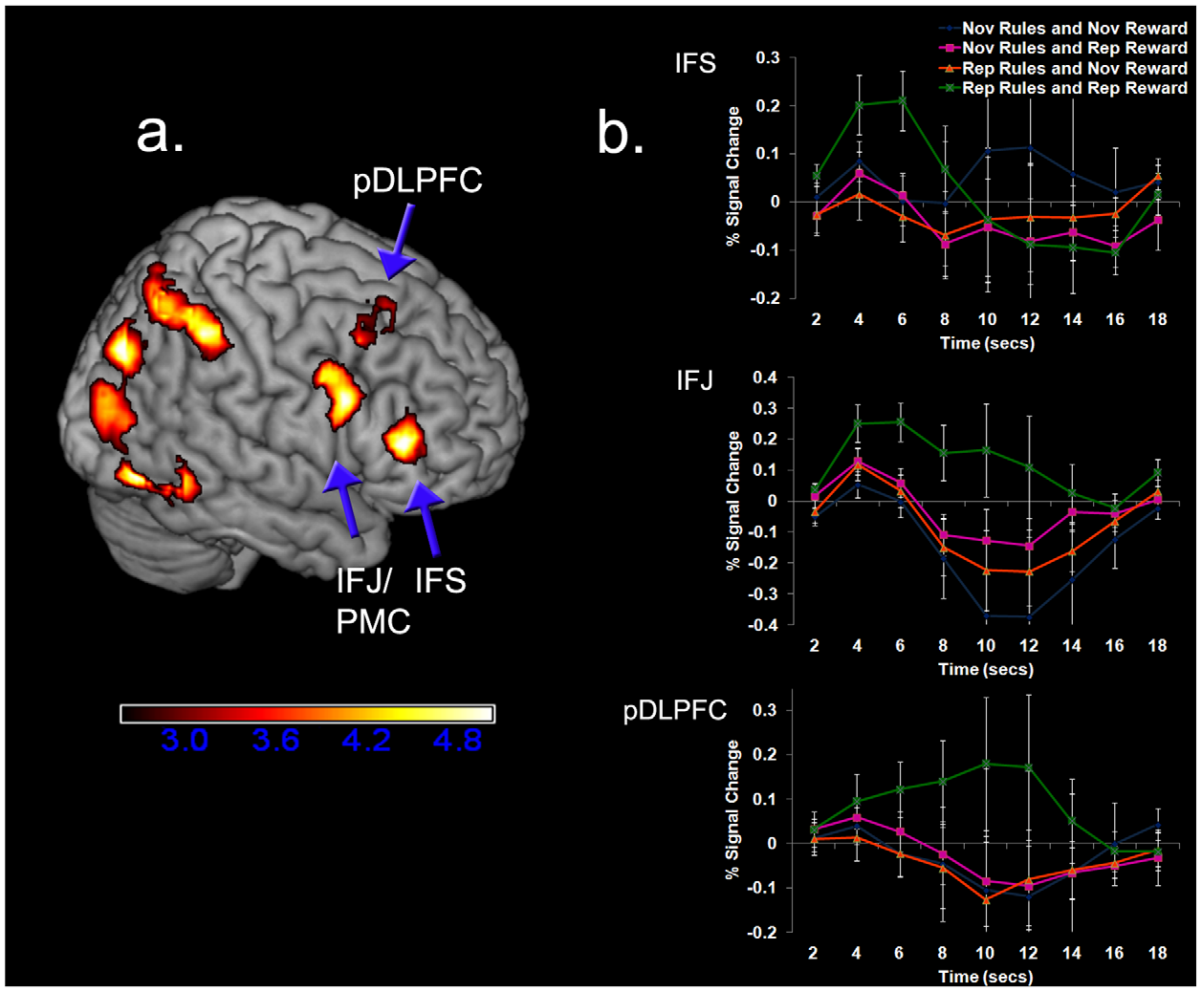
Regions Showing Repetition Enhancement for Repetition of a Specific Rule-Outcome Pairing (*Z*>2.57, *p*<.05 FWE cluster corrected for the whole-brain). **A.** Right lateral view showing regions of the LPFC exhibiting this effect. **B**. Activation time-course for the IFS, IFJ, and posterior DLPFC time-locked to the onset of the second instruction. The color scale denotes t-values. Nov = novel, rep = repeated. Error bars represent one (within-subject) standard error of the mean based on Loftus and Masson [69].

To examine our main question of whether the LPFC supports rule-outcome associations, we looked for an interaction between the rule and reward outcome factors, i.e., fMRI-adaptation when there is repetition of a specific rule-outcome pairing. Three areas within the right LPFC demonstrated an interaction in the form of repetition enhancement: 1) the inferior frontal sulcus (IFS; ∼ BA 45/46) extending onto the adjacent inferior and middle frontal gyri; 2) the pre-motor cortex (PMC) extending into the inferior frontal junction (IFJ; ∼BA 44/6); 3) the posterior dorsolateral prefrontal cortex (pDLPFC; ∼BA 8) (Figure 3a and Table 1; *Z*>2.57, *p*<.05 FWE cluster-corrected). The time-courses extracted from these regions (Figure 3b) demonstrated that activation increased for a repeated rule-outcome pairing relative to the novel rule-outcome pairing condition. Importantly, our exclusive masking analysis (see Materials and Methods) ensured, and visual inspection of the timecourse confirms, that these regions were not sensitive to repetition of the rules alone, or repetition of the reward outcome alone. Thus, these right LPFC areas are not showing an additive effect, but rather, are uniquely sensitive to specific rule-outcome associations. There were no behavioral differences across conditions [reaction time: *F*(3, 42) = 1.232, *p* = .31; accuracy: *F*(3, 42) = 1.196, *p* = .32], ruling this out as a confounding influence.

**Table 1:** Regions exhibiting fMRI-adaptation for repetition of rules and reward.

			*MNI coordinates*			
*Region*	*Hemisphere*	*BA*	*X*	*Y*	*Z*	*Z-score*
*Repeated rules and reward > novel rules and reward (repetition enhancement for rules and reward)*						
IFS	Right	45/46	48	36	12	4.72
PMC	Right	6/44	51	12	27	4.82
IFJ	Right	6/8/44	42	6	33	4.49
pDLPFC	Right	8	33	15	45	3.89
aIPS	Right	40	54	−42	54	4.78
vIPS	Right	7/19	33	−78	36	4.73
FFG	Right	19	39	−60	−12	5.09
MOG	Left	18	−33	−87	18	3.86
*Repeated rules and reward < novel rules and reward (repetition suppression for rules and reward)*						
Cerebellum	Right		36	−60	−51	6.26

Reported regions are significant at *Z*>2.57, *p*<.05 FWE cluster corrected for the whole-brain volume (k>112). BA = Brodmann area. IFS = inferior frontal sulcus; PMC = pre-motor cortex; IFJ = inferior frontal junction; pDLPFC = posterior dorsolateral prefrontal cortex; aIPS = anterior intraparietal sulcus; vIPS = ventral intraparietal sulcus; FFG = fusiform gyrus; MOG = middle occipital gyrus.

#### The IFS is Functionally Connected to Rule and Reward Regions

**Figure 4:**
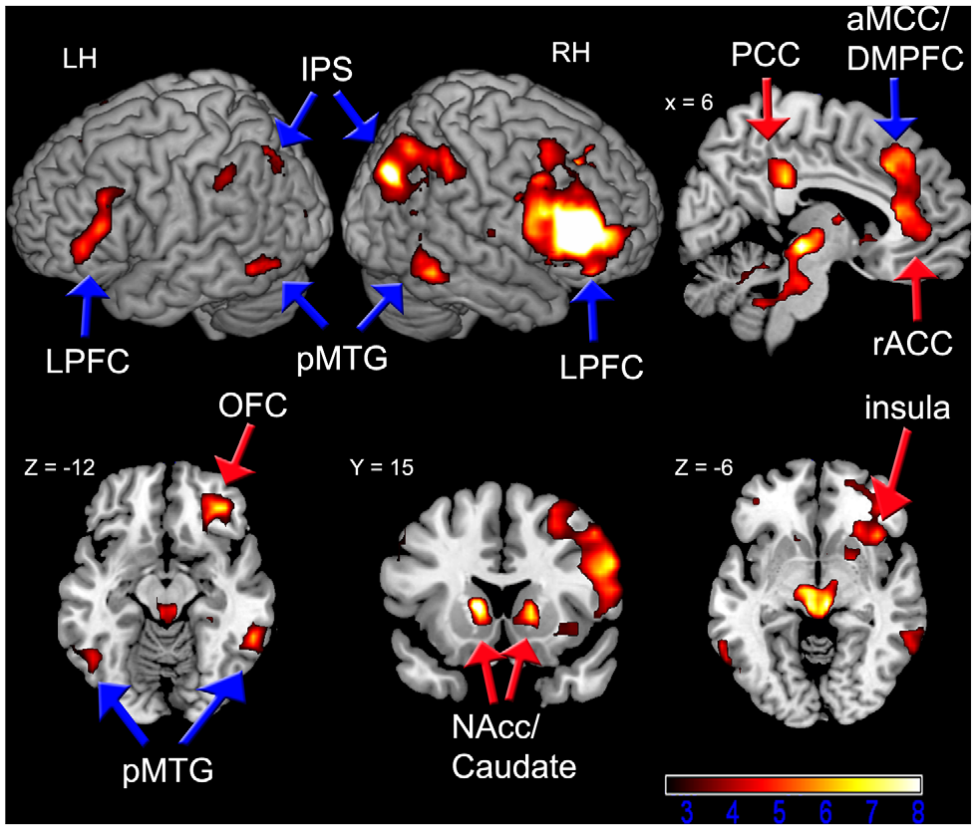
Regions Exhibiting Functional Connectivity with the IFS Across the Entire Time-Course (*Z*>2.57, *p*<.05 FWE cluster corrected for the whole-brain). Rule areas (blue arrows) include bilateral lateral prefrontal cortex (LPFC), anterior mid-cingulate cortex/dorsomedial prefrontal cortex (aMCC/DMPFC), posterior middle temporal gyrus (pMTG), and intraparietal sulcus (IPS). Reward areas (red arrows) include rostral anterior and posterior cingulate cortices (rACC, PCC), orbitofrontal cortex (OFC), caudate/nucleus accumbens (NAcc), and insula. The color scale denotes t-values, and the numerical values above the images correspond to MNI coordinates. For axial and coronal slices, the right hemisphere is on the right side of the image. LH = left hemisphere, RH = right hemisphere.

Based on the anatomical connections of the LPFC and the hypothesized associative function, we predicted that the LPFC would exhibit tonically coupled activity with rule related and outcome related regions. Consistent with this idea, and reinforcing the fMRI-adaptation findings, we found that the LPFC peak in the IFS exhibited significant functional connectivity with both rule and reward outcome processing regions across the entire experimental time-course (Figure 4 and Table 2; *Z*>2.57, *p*<.05 FWE cluster-corrected). IFS activation was significantly correlated with activation in rule processing areas including bilateral frontoparietal and lateral temporal cortices, as well as the aMCC, and cerebellum. Additionally, IFS activation was significantly correlated with activation in reward outcome processing areas including the rACC, bilateral caudate/NAcc, PCC, right OFC, and bilateral anterior insula. This pattern of connectivity is consistent with the idea that the IFS supports associations between rules and reward outcomes. Notably, these correlations probably reflect both spontaneous fluctuations and task-based influences (i.e., the functional requirement of processing rules and motivational outcomes).

**Table 2:** Regions exhibiting significant functional connectivity with the IFS.

			*MNI coordinates*			
*Region*	*Hemisphere*	*BA*	*X*	*Y*	*Z*	*Z-score*
pIFG/IFJ/ventral PMC	Right	44/6	57	18	27	5.03
RLPFC	Left	10/45	−54	51	0	4.57
IFS/IFG	Left	45/46	−48	38	15	4.03
Mid-cingulate/DMPFC	Medial	6/8/32	9	24	39	4.85
vIPS	Right	7/19	36	−75	39	5.52
aIPS	Right	40	57	−36	51	3.98
IPS	Left	7/39	−27	−66	45	3.60
aIPS/IPL	Left	7/40	−48	−39	42	3.31
pMTG	Right	21/37	57	−45	−12	4.36
pMTG	Left	21/37	−63	−54	−9	4.21
FFG/PHG	Left	20	−36	−36	−24	4.12
FFG/PHG	Right		36	−18	−27	3.46
Cerebellum	Right		12	−39	−33	3.80
rACC	Medial	24/32	−9	39	6	4.46
rACC	Medial	24/32	12	45	0	4.45
OFC	Right	11/13	36	39	−12	4.78
PCC	Medial	23/31	6	−33	33	4.40
Nacc/Caudate	Left		−12	3	9	5.63
NAcc/Caudate	Right		15	15	3	4.54
GP/Putamen	Right		21	6	−3	4.14
Mid-Brain	Right		−6	−18	−6	4.66
Anterior Insula	Right		39	21	−6	4.10
PAG	Right		6	−24	−6	5.00
PAG	Left		−6	−24	−6	4.73
Thalamus (MD)	Right		9	−12	0	4.13

Reported regions are significant at *Z*>2.57, *p*<.05 FWE cluster corrected for the whole-brain volume (k>106). BA = Brodmann area. vIPS = ventral intraparietal sulcus; aIPS = anterior intraparietal sulcus; pIFG = posterior inferior frontal gyrus; IFJ = inferior frontal junction; PMC = premotor cortex; DMPFC = dorsomedial prefrontal cortex; IFS = inferior frontal sulcus; pMTG = posterior middle temporal gyrus; FFG = fusiform gyrus; PHG = parahippocampal gyrus; IPL = inferior parietal lobule; NAcc = nucleus accumbens; PAG = periacqueductal gray; OFC = orbitofrontal cortex; rACC = rostral anterior cingulate cortex; VMPFC = ventromedial prefrontal cortex; PCC = posterior cingulate cortex; GP = globus pallidus; MD = mediodorsal nucleus.

## Discussion

Modern human life frequently requires cognitive control―selecting behaviors deliberately based on task rules held in work memory and suppressing automatic, inappropriate responses. What determines whether an individual will engage cognitive control at any given time? Across three different tasks―Stroop, WCST, and Go/No-Go―we found evidence that this decision is driven by expected motivational outcomes. That is, subjects' decision to select a response requiring active maintenance of the task rules depended on whether it was expected to yield a larger monetary reward than a pre-potent response. Importantly, there was no opportunity for trial-and-error learning of response value, suggesting that decisions were based on an *explicit* representation of expected outcomes. Self-reports collected after the experiment confirmed that subjects were aware of their choices and associated outcomes. Moreover, our results cannot be explained by a strategy in which subjects tended to exploit the automatic response and occasionally explore the cognitive control response; computational modeling has demonstrated that the decision to explore is largely driven by uncertainty and acquisition of new information [45], whereas in our task subjects were fully aware of the reward outcomes for each option, and therefore, had no reason to explore.

Despite the intuitive nature of the idea that the decision to engage cognitive control is driven by desired motivational outcomes, a dominant paradigm has been understanding reflective, rule-based behavior in terms of strictly cognitive mechanisms: reasoning, social standards, self-monitoring, and willpower [5], [6], [7]. Desire and motivation have been seen as obstacles for self-regulation. However, this idea overlooks the fact that individuals will only deliberately regulate their behavior if they are motivated to do so [8], [9]. Thus, in contrast to the perspective that successful self control is based on cold cognitive mechanisms overriding desires, we suggest that it is based on selecting the “proper” motivational incentive to guide action (often an incentive with long-term as opposed to immediate value). In other words, we suggest that decision making about rule-based behavior conforms to standard mechanisms of value-based decision making. The decision to follows rules at school or in the workplace and the decision to eat a chocolate bar may not be so different—both are driven by expected motivational outcomes, although the particular outcomes guiding those actions may be quite different. One implication of our findings is that promoting cognitive control may be more successful if time is taken to explicitly acknowledge the relationship between rules and outcomes. It would be useful for future work to directly compare the efficacy of different types of reward outcomes in motivating cognitive control and to investigate whether this pattern changes across the lifespan.

Decisions are not driven by expected reward magnitude alone, but factor in additional variables including probability of reward occurrence, required effort expenditure, and delay until the reward [46], [47], [48], [49]. It is suggested that these variables are integrated together resulting in an overall subjective “decision value”. Interestingly, we found that the tendency to engage cognitive control differed across our three behavioral tasks, being lowest for the Stroop task—the hardest task based on anecdotal reports. If the Stroop task required greater effort, this would have resulted in a lower decision value for engaging cognitive control relative to the easier tasks, and consequently, greater selection of the automatic response. Moreover, within each task, subjects almost invariably selected the automatic response when it was expected to yield a reward of equal magnitude to the cognitive control based response. This is again consistent with the idea of decision value, in that active maintenance of rules is generally assumed to require greater effort than a pre-potent response. Thus, our findings are consistent with the idea that subjects were integrating expected rewards with effort costs, however, future studies are necessary to directly examine this proposal.

Complementing the behavioral data, our fMRI-adaptation findings provide the first direct evidence that the lateral prefrontal cortex (LPFC) supports associations between rules and expected motivational outcomes. Several areas within the right LPFC including the inferior frontal sulcus (IFS) exhibited repetition enhancement uniquely when there was repetition of a specific rule-outcome pairing, but not when there was repetition of the rules alone or the expected outcome alone. Although repetition suppression is the more commonly observed form of fMRI-adaptation, repetition enhancement has also been demonstrated in numerous studies [37], [50], [51], [52], [53], , and may reflect a process of reinforcing top-down expectations for particular information [52], [55]. Irrespective of the precise interpretation of repetition enhancement effects, the important point is that it is believed to reflect the representation of specific information, and in our case, the effect in the LPFC was selective to repetition of a specific rule-outcome pairing. Moreover, strong corroborating evidence was provided by a functional connectivity analysis showing that the LPFC (specifically the IFS) exhibited correlated activity with both rule and reward related regions, consistent with this region serving as an integrative hub, linking rule and reward information. Our findings are additionally consistent with the anatomical connections of the LPFC [22] and recent work demonstrating activation near the right IFS during reward-based decision making [29], [30], [31] and during cognitive control engagement when monetary rewards are available [10].

Our findings are consistent with the idea that the LPFC actively maintains in working memory a representation of rules and the likely outcomes that will result from of implementing those rules. Creating such ‘online’ associations would allow the reward-value of *specific* rules to be rapidly updated when the state of the environment changes. This explicit rule-outcome associative mechanism for promoting cognitive control is consistent with the idea of a model-based learning system [56] and may operate in parallel with other (model-free) mechanisms. For example, O'Reilly and Frank [57] suggest that the mid-brain/basal ganglia system uses trial-and-error reinforcement learning to gradually “teach” the LPFC about information that should be held in working memory. This process would be akin to forming a habit to engage cognitive control. This mechanism may be efficient for engaging cognitive control in stable, familiar environments after learning has taken place. In contrast, explicit associations between rules and expected rewards may dominate in novel or rapidly changing environments, and situations in which overt reward feedback is not provided and there is no possibility for dopamine mediated reinforcement learning.

It is also useful to consider our findings in relation to performance monitoring theories [16], [58], [59]. These theories suggest that detection of motivationally salient events by the MPFC including response conflict, rewards, and errors, will result in a modulatory gain signal that is sent to the LPFC to influence the strength of cognitive control. This account provides a parsimonious mechanism by which motivational events could dynamically alter the strength of an *already activated* rule. It does not however, directly address how a rule is initially selected to guide action. By definition, a modulatory gain signal sent by the MPFC will simply amplify whatever is already being represented by the LPFC. Our findings together with this model suggests the following: a specific rule is initially selected to guide action based on the explicit expectation that using the rule will lead to a desirable outcome (mediated by the LPFC), and then the strength of the selected rule is optimized according to ongoing performance and events in the environment (mediated by MPFC-LPFC interactions).

A widespread heuristic for understanding the organization of the prefrontal cortex is that the LPFC supports complex cognitive processes (including rule-use) whereas orbital and medial prefrontal areas support motivational processes. One problem with this perspective is that it is largely predicated on studies that have conflated task difficulty with presence of motivational incentives. In general, studies examining motivational processing have used simple tasks, while studies of complex cognition have rarely incorporated rewards (or other motivational incentives). Recent work has started to depart from this methodological trend, and has revealed LPFC involvement in numerous aspects of complex motivational processing: decision making involving risk [31], [32], integrating multiple variables (e.g., reward magnitude and delay) to discern the best course of action [26], [30], monitoring action-outcome history during strategic decision making [33], representing the long-term motivational context (i.e., what reward is likely to occur over many trials) [24], and specific decision making strategies [34]. Moreover, recent work has demonstrated that cognitive control related activity in the LPFC is modulated by the availability of reward incentives [10], [11], [14], [15], [17]. Finally, our findings directly suggest that the LPFC has a role in complex motivational processing by representing the relationship between specific rules for action and expected motivational outcomes. This idea is a natural extension of work highlighting the role of the orbitofrontal cortex in representing object-outcome associations [2], [3], [4], and the role of the cingulate cortex in representing action-outcome associations [60], [61]. In sum, it is clear that the LPFC has a more direct role in motivational processes than traditionally recognized.

Some methodological considerations are worth noting. We designed our fMRI task to minimize the impact of extraneous factors that might account for any fMRI-adaptation effect we might observe. Importantly, we held constant the content of the second instruction cue allowing us to examine neural activation in response to the identical event, simply as a function of how it was primed by the first instruction cue. This ensures that the fMRI-adaptation effects we observed cannot be explained by differences across conditions in visual processing or interpretation of the second instruction cue, or differences relating to expectation of the ensuing stimulus. Furthermore, given that there was never repetition of visual information across the first and second instruction cue, we can be sure that fMRI-adaptation was related to repetition of the conceptual representation of the rules and expected reward and not to repetition of the visual symbols used to signal this information. Additionally, it is unlikely that our findings are due to differences across conditions in difficulty, attention, or effort. It could be argued that when the second instruction cue signals repeated rules and reward, the task is easier, and subjects don't really need to process or attend to the second instruction cue, and therefore, may engage in mind-wandering—and that this led to the increased activations (i.e., the repetition enhancement effect) that was selective to this condition. This is very unlikely for two reasons: 1) increased activation was not observed in medial prefrontal and parietal regions that are consistently associated with mind-wandering and 2) mind-wandering is often associated with poorer performance, however, we found that performance was equivalent across conditions. Additionally, in the unlikely case that the repeated rule-reward pairing condition was more difficult and required greater effort or attention, it would be expected to result in a *quantitative* difference in activation levels across conditions (akin to a gain enhancement effect). However, we found a *qualitatively* different pattern of activations; the repetition enhancement effect was observed after masking out all voxels that were sensitive to repetition of the rules alone or repetition of the reward alone.

In summary, our findings suggest that individuals will engage cognitive control when they see the *value* in doing so―that is, when rule-use is expected to yield a desired outcome. The LPFC contributes to this process by representing associations between rules and expected outcomes, and performs this associative function within the context of interactions with widely distributed rule and reward related regions. An implication of our findings is that individual differences in cognitive control may result not only from the capacity to represent rules, but also the capacity to discern (or focus on) motivationally significant outcomes that will result from rule-use. Finally, our findings and several previous studies [39], [40], [41] highlight the feasibility of using fMRI-adaptation to examine complex cognitive and motivational processes.

## Materials and Methods

The study was approved by the UBC behavioral and clinical research ethics boards.

### Participants

Participants were 16 healthy adults (*M* = 26.6, *SD* = 3.4; 8 female) in experiment 1 and 15 (right-handed) healthy adults (*M* = 27.4, *SD* = 5.51; 8 female) in experiment 2, with no history of psychiatric or neurological illness, and all provided written informed consent and received payment for their participation.

### Behavioral Task Paradigm

All three tasks involved the same structure. First, there was a short practice block in which subjects had several trials to become familiarized with responding to the stimuli in the two different ways (e.g., responding based on the word meaning and based on the ink colour in the Stroop task). Second, in the training phase, a habit (i.e., an automatic, pre-potent response) was established by having subjects repeatedly respond to the stimuli in one way (Stroop: identify the word meaning; WCST: match based on color; Go/No-Go: respond to every letter including ‘X’). Subjects performed a minimum of 100 trials and moved on to the next phase once they reached an average of 80% accuracy across the last 20 trials. Finally, in the free-choice phase, subjects were given the option of responding based on the habit, or based on the alternative way to respond to the stimulus, which required cognitive control, i.e., an active representation of the rules to overcome the habit (Stroop: identify the ink color; WCST: match based on shape; Go/No-Go: respond to every letter *except* ‘X’). Prior to each mini-block of four trials during the free-choice phase, a screen appeared and indicated the amount of money that could be earned if the subject chose the habit or engaged cognitive control (e.g., ink color = 25¢, word meaning = 50¢). Subjects were told that they would have to respond correctly on four consecutive trials to earn the money. (in reality all subjects were paid $12 at the end of the experiment.) Following completion of a four trial mini-block, the next monetary value screen appeared, and subjects were free again to choose how to respond. There were 5 conditions, defined by the following monetary amounts: 25¢/25¢ (equal value); 50¢/25¢ (habit value greater by a small amount); $1.00/25¢ (habit value greater by a large amount); 25¢/50¢ (cognitive control value greater by a small amount); 25¢/$1.00 (cognitive control value greater by a large amount). Subjects saw each condition twice in random order. Thus, in total, subjects performed 40 free choice trials. No feedback was provided after choices, precluding trial-and-error learning of response value. Rather, subjects could only use an *explicit representation* of the expected monetary amounts to guide their decisions. The order of the Stroop, WCST, and Go/No-Go task was random, and for each of task, stimulus order was random with the constraint that within each mini-block, no particular stimulus appeared more than once.

#### Stroop Task

During the training phase, on every trial subjects saw the word red or the word blue, written in red or blue ink, and pressed one of two buttons to indicate the *word meaning*. The ink colour could be congruent or incongruent with the word meaning. During the free-choice phase, the stimuli were always incongruent and subjects were free to respond based on the word meaning (habit response) or the ink color (cognitive control response). The incongruent stimuli allowed us to discern which response type the subject was choosing during each mini-block. For example, if it was the word red written in blue ink, and if the subject indicated red, that would mean that they identified the word meaning, whereas if they indicated blue, that would mean that they identified the ink colour. Stimulus duration was 750 ms and 1200 ms for the training and free-choice phases, respectively. A fixation cross appeared after each stimulus for 1000 ms.

#### Wisconsin Card Sorting Task (WCST)

Our simplified version of the WCST involved just two dimensions, color and shape. On each trial, subjects saw three coloured-shapes: one presented in the upper visual field served as the target and the other two presented in the lower visual field served as the choice stimuli. Each choice stimulus matched the target on one dimension (color or shape). During the training phase, subjects indicated the location (right or left side of the screen) of the choice stimulus matching the target on the *color* dimension. During the free-choice phase, subjects were free to choose: they could indicate the side of the screen containing the stimulus matching in color (habit response) or the side of the screen containing the stimulus matching in shape (cognitive control response). Thus, the cognitive control response required an extra-dimensional shift. Given that each choice stimulus matched the target on one dimension, we could discern which response type they were selecting. The stimuli were triangles or hexagons and were orange or green. Stimulus duration was 1000 ms and 1400 ms for the training and free-choice phases, respectively. A fixation cross appeared after each stimulus for 1000 ms.

#### Go/No-Go task

On each trial one of four letters (A, G, T, X) was presented and subjects made a button press as soon as possible after appearance of the letter. During the training phase, subjects responded to all letters *including* ‘X’. During the free-choice phase, subjects could continue responding to all letters (habit response), or they could respond to all letters *except* for ‘X’, i.e., inhibit responding when the ‘X’ was presented (cognitive control response). Stimulus duration was 350 ms for both the training and free-choice phases. A fixation cross appeared after each stimulus for 500, 1000, or 1500 ms (duration selected randomly among these values). The jittered ISI made the task more difficult and more firmly established a habit to make a response any time a letter appeared on the screen.

#### Data Analysis

For each of the five monetary value conditions, we calculated the percentage of choices during which the cognitive control response was selected. To confirm that subjects were making decisions based on explicit knowledge of the reward outcomes, we also collected self-report data following the experiment; subjects explicitly noted their *intended* response as a function of the expected reward amount presented during the task. We found 93% correspondence between observed behaviors and self-reported intended responses.

To compare reaction times (RTs) for the habit-based responses and cognitive control-based responses, we analyzed median RTs with paired-samples t-tests (we collapsed across reward amount conditions, due to the fact that in many cases, a given condition only elicited one response type; e.g., the automatic response was almost always chosen for the $1.00/25¢ condition). Given our strong hypothesis that the cognitive control response would be slower, directional (one-tailed) p-values are reported.

### fMRI-Adaptation Paradigm

The software package E-prime (Psychology Software Tools, Pittsburg, PA, USA) was used to implement the task. Stimuli were presented using a back-projection system. The trial structure of the fMRI-adaptation paradigm is illustrated in Figure 2. On each trial, subjects performed one of two tasks: decide if a face is male/female or decide if a word's meaning is concrete/abstract. These tasks required simple if-then rules (e.g., if faces task and if male then press button “1”, if female then press button “2”). Prior to stimulus presentation, an instruction cue informed subjects of the relevant rules to use and whether or not to expect a monetary reward (25¢ contingent on correct performance). Subjects received $30 for participating in the fMRI scanning session and were told that they could earn an additional $30 if they earned all of the available money. The instruction cues were familiar visual images that subjects learned prior to the experiment and were selected to be easy to represent in mind (see Figure 2). The instruction cues did not specify a particular response, but rather, a set of stimulus-response contingencies (i.e., rules). Subjects were told to explicitly think about the rules and expected outcome signaled by each instruction cue. Following presentation of the instruction cue, there was a delay period followed by presentation of a word or face stimulus. During this time, subjects made a button-press response. Subsequently, a screen indicated cumulative monetary winnings and whether or not money had been earned on that trial.

On 40% of trials, a single instruction cue appeared prior to the stimulus. Crucially, on the remaining 60% of trials, the first instruction cue was followed by a delay period and then presentation of a second instruction cue. On these double-instruction cue trials, subjects were told to forget the first instruction cue and to respond to the stimulus based on the content of the second instruction cue. These double-instruction cue trials allowed us to examine fMRI-adaptation.

Given that nearly half of the trials were single cue trials and subjects were explicitly instructed to avoid expecting a second cue, this ensured that subjects paid attention to the first cue. Each rule and expected outcome (money versus no money) was represented with two different visual images. During repetition of the rules, expected reward, or both, two distinct visual images were used so that there was never repetition of the visual features of the cue, but only its symbolic meaning.

Subjects performed 162 trials in total. There were 96 were double-instruction cue trials in which each of the four key conditions noted above appeared 24 times. There were 66 single-instruction cue trials. There were 12 repetitions of each of the rule-outcome combinations: male/female rules + no reward; male/female rules + reward; abstract/concrete rules + no reward; abstract/concrete rules + reward. The other 18 single-cue trials were additional male/female rules + no monetary reward trials, which ensured that 40% of the trials were single-cue trials, 25% were neutral (no monetary reward), and 25% presented the male/female rule. Trials were presented pseudorandomly such that double-cue trials never occurred more than twice in a row and no condition appeared more than twice in a row.

Trials began with a jittered interstimulus interval (mean = 4.9 s, range = 2–7.5 s, increments of 500 ms), followed by presentation of the first instruction cue (2 s). This was followed by a variable length delay (mean = 5 s; range = 4–6 s; increments of 1000 ms). Next the word or face stimulus appeared (2 s) during which time subjects made their response. Finally a reward screen (1.5 s) revealed to subjects their total current winnings and also if they earned money on that trial. On some trials, a second instruction cue (2 s) appeared followed by a delay (4 s) prior to stimulus presentation. Given the delay length of 4, 5, or 6 s before the key event of interest (i.e., instruction cue 2), this allowed us to effectively estimate the BOLD response separately for the first and second instruction cues and also provided a temporal resolution of 1000 ms with respect to sampling the hemodynamic response function.

Given the demanding nature of the task, we included a rest period of 15 s (filled with a blank screen) in middle of each session to provide subjects with a brief break. A blank screen also appeared for 10 s at end of each session to allow the BOLD response to return to baseline. Additionally, one day prior to scanning, subjects came in for a one-hour training session. Subjects learned the correspondence between the instruction cue visual images and their meaning and then received 80 practice trials. Although our task was difficult, this training ensured that during the scanning session, subjects were able to effectively process the information signaled by each instruction cue.

#### Stimuli

The words were chosen from the Medical Research Council Psycholinguistic Database (http://www.psy.uwa.edu.au/mrcdatabase/uwa_mrc.htm). The words had a minimum of three letters and a maximum of eight letters, and a minimum written frequency of 30. Words selected for the “concrete” category (e.g., bag), had a concrete rating above 600 and words selected for the “abstract” category (e.g., advice) had a concrete rating below 300. The face stimuli were high resolution front-view photographs of neutral expression faces obtained from several image databases [62], [63], [64]. In total, 42 photographs (21 male, 21 female) were selected. The faces were cropped to remove hair and other non-facial features, gray-scaled, equated in size, and then we added 10% Gaussian noise to increase the difficulty of the face discrimination (making it more comparable to the abstract/concrete discrimination). Stimuli subtended 4.5 (width) ×4.7 (height) degrees visual angle.

#### fMRI Data Acquisition

fMRI data were collected using a 3.0-Tesla Philips Intera MRI scanner (Best, Netherlands) with a standard 8-element 6-channel phased array head coil with parallel imaging capability (SENSE). Head movement was restricted using foam padding around the head. T2*-weighted functional images were acquired parallel to the anterior commissure/posterior commissure (AC/PC) line using a single shot gradient echo-planar sequence (repetition time, TR = 2 s; echo time, TE = 30 ms; flip angle, FA = 90°; field of view, FOV = 24×24×14.3 cm; matrix size = 80×80; SENSE factor = 1.0). Thirty-six interleaved axial slices covering the whole brain were acquired (3-mm thick with 1-mm skip). Data collected during the first 4 TRs were discarded to allow for equilibration effects. There were six sessions approximately 9-minutes long each during which 1608 volumes were acquired in total.

After functional imaging, in-plane inversion recovery prepared T1-weighted anatomical images were acquired in the same slice locations as the functional images using a fast spin-echo sequence (TR = 2 s; TE = 10 ms; 36 interleaved axial slices covering the whole brain, 3-mm thick with 1-mm skip; FA = 90°; FOV = 22.4×22.4×14.3 cm; matrix size = 240×235; reconstructed matrix size = 480×470; inversion delay = 800 ms; spin echo turbo factor = 5).

#### fMRI Data Preprocessing

Image preprocessing and analysis were conducted with Statistical Parametric Mapping (SPM5, University College London, London, UK; http://www.fil.ion.ucl.ac.uk/spm/software/spm5). The time series data were slice-time corrected (to the middle slice), realigned to the first volume to correct for between-scan motion (using a 6 parameter rigid body transformation), and coregistered with the T1-weighted structural image. The in-plane T1 image was bias-corrected and segmented using template (ICBM) tissue probability maps for gray/white matter and CSF. Parameters obtained from this step were subsequently applied to the functional (re-sampled to 3 mm^3^ voxels) and structural (re-sampled to 1 mm^3^ voxels) data during normalization to MNI space. The data were spatially-smoothed using an 8-mm^3^ full-width at half-maximum Gaussian kernel to reduce the impact of inter-subject variability in brain anatomy. Finally, a linear detrending procedure [65] was applied to remove time-series components that were correlated with global changes in the BOLD signal.

#### fMRI Data Analysis: First-Level Model

Data were analyzed at the first level with a general linear model. There were 19 key regressors that were convolved with a synthetic hemodynamic response function. Four regressors modeled as delta (stick) functions coded the information contained in the first instruction cue: (1) male/female rules and no monetary reward, (2) male/female rules and monetary reward (3) abstract/concrete rules and no monetary reward, (4) abstract/concrete rules and monetary reward. Four regressors modeled as variable-duration (4–6 s) epochs coded the subsequent delay period following each of these events. Four regressors modeled as delta (stick) functions coded the second instruction cue―which was always the identical event (abstract/concrete rules and monetary reward)―as a function of how it was primed by the preceding instruction cue: (1) novel rules and novel reward, (2) repeated rules and novel reward, (3) novel rules and repeated reward, and (4) repeated rules and repeated reward. Four regressors modeled as 4 s fixed-duration epochs coded the subsequent delay period after these events. Additional regressors modeled as delta functions coded presentation of the stimulus and reward screen, and a regressor modeled as a variable-duration (10 or 15 s) epoch coded the rest period at the middle and end of each session. The model also included the six movement parameters estimated during realignment, and regressors coding session effects. Serial autocorrelations were modeled using AR(1) and the data were high-pass filtered (1/128 Hz) to remove low frequency drift in the BOLD signal. Given that performance was at near ceiling levels, modeling correct and incorrect responses had a negligible effect, so they were left out in order to simplify the model.

To examine neural activation during the second instruction cue, we created four contrast images to capture each of the conditions: (1) novel rules and novel reward > fixation, (2) novel rules and repeated reward > fixation, (3) repeated rules and novel reward > fixation, (4) repeated rules and repeated reward > fixation.

#### Second-Level Random Effects Analysis

The contrasts created for each subject were subsequently submitted to a group level analysis, a 2 (rules: novel versus repeated) ×2 (reward: novel versus repeated) factorial ANOVA. To isolate regions sensitive to rules alone or the expected reward alone, we examined the main effects. To probe whether the LPFC is especially sensitive to specific rule-reward pairings we looked for a specific interaction between the rule and reward factors in the form of: repeated rules and repeated reward < novel rules and repeated reward = repeated rules and novel reward = novel rules and novel reward. This would correspond to repetition suppression for repeated rule and reward information, and the inverse of this contrast would correspond to repetition enhancement for repeated rule and reward information. This interaction was captured with the contrast weights of: −3 1 1 1 and 3 −1 −1 −1 (for repetition suppression and enhancement, respectively) [66], [67]. Note that these contrast weights assess the interaction in a valid form that is meaningful for our theoretical question, and does not assess the traditional “cross-over” interaction.

To provide a stringent test of the hypothesis that regions showing an interaction effect were *selective* to repetition of a specific rule-reward pairing and not sensitive (even weakly) to repetition of the rules alone or the reward alone, we used an exclusive masking analysis. We excluded voxels demonstrating fMRI-adaptation (repetition enhancement or suppression) for repetition of rules alone, or reward alone at a very lenient threshold (*p*<.05 uncorrected), and then we looked for regions demonstrating fMRI-adaptation for repetition of the rule-reward pairing condition (at *Z*>2.57, *p*<.05 FWE corrected). The lenient threshold for the voxels being masked out made this a very conservative analysis with respect to finding regions that exhibit adaptation selectively for repeated rule-reward pairings. Moreover, the masking analysis ensured that we were not simply identifying regions showing an additive effect of rule and reward processing. To identify rule-selective and reward-selective voxels to be masked out, we used simple effect contrasts rather than main effect contrasts because the main effect contrasts include the repeated rules-repeated reward condition within their computation¯the specific condition we were interested in identifying.

#### Correction for Multiple Comparisons

To create maps of significant effects, we used a cluster-forming threshold of *Z*>2.57 (*p*<.005 uncorrected), and corrected for multiple comparisons using family-wise error (FWE) correction for cluster extent (*p*<.05) based on random field theory. Correction for multiple comparisons was calculated based on the whole brain volume and corresponded to a cluster size of between 106–112 voxels, depending on the specific analysis (adaptation effects or functional connectivity).

#### Time-Course Visualization

To visualize the time-course of the LPFC regions showing an interaction effect, we used the Marsbar toolbox in SPM5 [68] (http://marsbar.sourceforge.net/) to extract average signal change values from 3-mm radius spheres for each subject centered on peak voxels from the group analysis. We used 12 finite impulse response (FIR) functions, one for each peristimulus time point within a trial window of 24 s following onset of the second instruction cue.

#### Functional Connectivity

We took the IFS time-course extracted for each subject scaled it by the mean global brain signal at each time point to minimize the effect of global drift, and then converted to percent signal change values by subtracting and diving by the mean value of the ROI for the appropriate session. The data were also high-pass filtered (1/128 Hz). The normalized time-course for each subject was then used as a regressor in a first-level GLM analysis that also included the six motion parameters obtained from realignment as covariates of no interest. We created contrast images for each subject assessing positive connectivity across the 6 functional sessions. These contrast images were then brought to a second-level random effects analysis and entered into a one-sample *t-test* to identify voxels across the brain showing a correlation with IFS that differed significantly from zero. Our functional connectivity analysis is very similar to standard approaches to analyzing resting state networks. However, in this case, our results will likely reflect both spontaneous fluctuations and task-related influences.

## Supporting Information

Table S1 **Regions exhibiting fMRI-adaptation for repetition of the reward.** Reported regions are significant at *Z*>2.57, *p*<.05 FWE cluster corrected for the whole-brain volume (k>112). BA = Brodmann area. PCC = posterior cingulate cortex; MD = mediodorsal nucleus; AN = anterior nucleus; IFG = inferior frontal gyrus; NAcc = nucleus accumbens; rACC = rostral anterior cingulate cortex; OFC = orbitofrontal cortex; pMTG = posterior middle temporal gyrus; IPS = intraparietal sulcus; aIPS = anterior intraparietal parietal sulcus; IPL = inferior parietal lobule; IFS = inferior frontal sulcus; OTC = occipitotemporal cortex; MOG = middle occipital gyrus.
(DOCX)

Table S2 **Regions exhibiting fMRI-adaptation for repetition of the rules.** Reported regions are significant at *Z*>2.57, *p*<.05 FWE cluster corrected for the whole-brain volume (k>112). BA = Brodmann area. pSTS = posterior superior temporal sulcus; aMCC = anterior mid-cingulate cortex; SMA/pre-SMA = supplementary motor cortex/presupplementary motor cortex; pMTG = posterior middle temporal gyrus; IPS = intraparietal sulcus; IPL = inferior parietal lobule; IFG = inferior frontal gyrus; IFJ = inferior frontal junction; PMC = premotor cortex; MOG = middle occipital gyrus; RLPFC = rostrolateral prefrontal cortex; pMFG = posterior middle frontal gyrus; lOFC = lateral orbitofrontal cortex; DMPFC = dorsomedial prefrontal cortex.
(DOCX)
