# Supplementary material for: The Decision to Engage Cognitive Control Is Driven by Expected Reward-Value: Neural and Behavioral Evidence
Source: PLoS One. 2012 Dec 19;7(12):e51637. doi: 10.1371/journal.pone.0051637 (PMC3526643; doi:10.1371/journal.pone.0051637)
Supplement: Table S1 — Regions exhibiting fMRI-adaptation for repetition of the reward. Reported regions are significant at Z>2.57, p<.05 FWE cluster corrected for the whole-brain volume (k>112). BA = Brodmann area. PCC = posterior cingulate cortex; MD = mediodorsal nucleus; AN = anterior nucleus; IFG = inferior frontal gyrus; NAcc = nucleus accumbens; rACC = rostral anterior cingulate cortex; OFC = orbitofrontal cortex; pMTG = posterior middle temporal gyrus; IPS = intraparietal sulcus; aIPS = anterior intraparietal parietal sulcus; IPL = inferior parietal lobule; IFS = inferior frontal sulcus; OTC = occipitotemporal cortex; MOG = middle occipital gyrus. (DOCX) [file pone.0051637.s001.docx]

|  |  |  | *MNI coordinates* | | |  |
| --- | --- | --- | --- | --- | --- | --- |
| *Region* | *Hemisphere* | *BA* | *X* | *Y* | *Z* | *Z-score* |
|  |  |  |  |  |  |  |
| *Novel reward > repeated reward (repetition suppression for reward)* | | | | | | |
|  |  |  |  |  |  |  |
| NAcc | Right |  | 12 | 9 | -3 | 4.67 |
| NAcc | left |  | -12 | 15 | -6 | 3.50 |
| rACC | Medial | 24/32 | 9 | 39 | 18 | 4.64 |
| PCC | Medial | 23 | -3 | 21 | 30 | 4.84 |
| Insula/caudal OFC | Left |  | -24 | 21 | -15 | 4.30 |
| Insula/caudal OFC | Right |  | 24 | 21 | -15 | 4.30 |
| Mid-Brain | Left |  | -6 | -15 | -15 | 3.97 |
| Thalamus (MD/AN) | Left |  | -6 | -15 | 12 | 4.69 |
| IFG (pars triangularis) | Left | 45 | -54 | 33 | 0 | 4.69 |
| Globus Pallidus | Right |  | 18 | 3 | -3 | 3.94 |
|  |  |  |  |  |  |  |
| *Novel reward < repeated reward (repetition enhancement for reward)* | | | | | | |
|  |  |  |  |  |  |  |
| PHG/FFG | Right | 37 | 30 | -45 | -12 | 5.86 |
| PHG/FFG | Left | 37 | -24 | -48 | -15 | 5.73 |
| pMTG | Right | 37/21 | 51 | -54 | -9 | 4.96 |
| pMTG | Left | 37 | -51 | -69 | 3 | 4.35 |
| Mid IPS | Right | 7/39 | 30 | -75 | 45 | 4.57 |
| Ventral IPS | Left | 19 | -30 | -84 | 33 | 4.42 |
| IFS | Right | 45 | 45 | 36 | 12 | 4.47 |
| OTC/MOG | Right | 19 | 45 | -81 | 18 | 4.04 |
| OTC/MOG | Left | 19 | -42 | -84 | 15 | 3.79 |
| aIPS/IPL/Somatosensory cortex | Right | 40/2 | 36 | -39 | 45 | 5.32 |
|  |  |  |  |  |  |  |
