# Supplementary material for: The Decision to Engage Cognitive Control Is Driven by Expected Reward-Value: Neural and Behavioral Evidence
Source: PLoS One. 2012 Dec 19;7(12):e51637. doi: 10.1371/journal.pone.0051637 (PMC3526643; doi:10.1371/journal.pone.0051637)
Supplement: Table S2 — Regions exhibiting fMRI-adaptation for repetition of the rules. Reported regions are significant at Z>2.57, p<.05 FWE cluster corrected for the whole-brain volume (k>112). BA = Brodmann area. pSTS = posterior superior temporal sulcus; aMCC = anterior mid-cingulate cortex; SMA/pre-SMA = supplementary motor cortex/presupplementary motor cortex; pMTG = posterior middle temporal gyrus; IPS = intraparietal sulcus; IPL = inferior parietal lobule; IFG = inferior frontal gyrus; IFJ = inferior frontal junction; PMC = premotor cortex; MOG = middle occipital gyrus; RLPFC = rostrolateral prefrontal cortex; pMFG = posterior middle frontal gyrus; lOFC = lateral orbitofrontal cortex; DMPFC = dorsomedial prefrontal cortex. (DOCX) [file pone.0051637.s002.docx]

|  |  |  | *MNI coordinates* | | |  |
| --- | --- | --- | --- | --- | --- | --- |
| *Region* | *Hemisphere* | *BA* | *X* | *Y* | *Z* | *Z-score* |
|  |  |  |  |  |  |  |
| *Novel rules > repeated rules (repetition suppression for rules)* | | | | | | |
|  |  |  |  |  |  |  |
| IFG (pars triangularis) | Left | 45 | -57 | 36 | -3 | 3.79 |
| SMA/pre-SMA | Medial | 6 | 0 | -3 | 54 | 3.97 |
| ventral S1/M1 | Left | 3/4 | -60 | -12 | 27 | 3.95 |
| vPMC/opercularis | Left | 6/44 | -54 | 3 | 39 | 3.65 |
| aMCC | Medial | 24/32 | -9 | 15 | 39 | 3.86 |
| pSTS | Left | 22/21 | -60 | -39 | 9 | 5.32 |
| STS | Right | 41/42 | 60 | -15 | 15 | 3.96 |
| pMTG | Left | 37 | -54 | -54 | 3 | 5.04 |
| pMTG | Right | 37 | 48 | -63 | 3 | 3.57 |
| Cerebellum | Right |  | 27 | -60 | -27 | 4.39 |
|  |  |  |  |  |  |  |
| *Repeated rules > novel rules (repetition enhancement for rules)* | | | | | | |
|  |  |  |  |  |  |  |
| RLPFC/lOFC | Left | 10 | -36 | 54 | -9 | 4.25 |
| pMFG | Left | 8 | -36 | 21 | 45 | 4.12 |
| IFJ/PMC/pMFG | Right | 6/44 | 42 | 12 | 42 | 3.93 |
| IPL | Right | 40 | 45 | -51 | 48 | 4.55 |
| IPL | Left | 40 | -51 | -54 | 39 | 3.85 |
| SMA | Right | 6 | 15 | 18 | 66 | 4.11 |
| DMPFC | Medial | 8 | -3 | 39 | 36 | 3.52 |
| MOG | Left | 18 | -12 | -99 | 12 | 4.38 |
| MOG | Right | 18 | 15 | -99 | 18 | 4.02 |
|  |  |  |  |  |  |  |
